# The lived experiences of woman navigating university with lupus and their primary support persons

**DOI:** 10.1177/09612033261461992

**Published:** 2026-06-17

**Authors:** Hailey A. O’Neil, Misha Behi, Paula C. Fletcher

**Affiliations:** 1 Department of Kinesiology and Physical Education, 8431 Wilfrid Laurier University, Waterloo, ON, Canada

**Keywords:** chronic disease, support, qualitative, healthcare, education

## Abstract

**Objective:**

This study examined the lived experiences of women with lupus and their primary support persons to better understand the effects associated with navigating university/college with lupus.

**Methods:**

Five women with lupus and five primary support persons (*n* = 10) were recruited from Southern Ontario. One-on-one semi-structured interviews were conducted with participants. Reflexive thematic analysis was used to analyze verbatim transcripts.

**Results:**

Two salient themes, (1) *Lupus is a part of me*, and (2) *Lupus does not exist in a bubble*, were identified, each with associated sub-themes. *Lupus is a part of me* discussed how lupus was part of participants everyday lives and how it became their new normal following symptom onset. *Lupus does not exist in a bubble* explored the ongoing challenges individuals and support persons experienced due to the lack of knowledge within healthcare, education, and society.

**Conclusions:**

Women with lupus who are navigating university endure unique challenges in addition to school-related demands. Similarly, primary support persons who assisted throughout this navigation period encountered several individual and situational hardships due to the nature of lupus. Education for healthcare professionals, universities, and individuals with lived experience is warranted, along with more support services, to help navigate university/college more effectively.

## Introduction

Women are disproportionally affected by lupus and account for 90% of all lupus cases.^1^ In addition to the symptoms associated with lupus, obtaining a diagnosis before the age of 18 leads to challenges within school,^2^ and transitions between pediatric and adult care.^3^ When examining the relationship between the effects of lupus and school, the majority of research has focused on children in elementary school,^2,4^ adolescents in high school,^2,4^ or adults with systemic lupus erythematosus reflecting on their education years retrospectively,^5^ with only one study exploring the effects of lupus on university or college-aged students.^6^ The transition into, and navigation through, university or college for any student can be psychologically demanding and can negatively influence mental health^7^; however, for students with chronic illnesses, such as lupus, the effects of these transitions and school-related demands may be exacerbated by their lupus and lack of support available.^8^ For example, a college student with lupus discussed the challenges associated with walking to/from classes, carrying her books and managing her course load due to her fluctuating lupus symptoms (e.g., fatigue). These symptoms also caused challenges socializing which led to feelings of isolation.^6^

Unfortunately, there is a paucity of information regarding women navigating university/college and the role of their primary support persons. Understanding their experiences will provide an in-depth understanding of the effects of lupus on students, which in turn may inform policy to increase support services while attending university/college. As such, the purpose of this study was to examine the lived experiences of students/recent graduates who are women with lupus navigating university/college, as well as their primary support persons. Specifically, this study aimed to uncover the effects associated with living and coping with lupus for both individuals with lupus and their primary support persons during this transitional period.

## Methods

### Design

The theoretical orientation which guided this study was interpretive phenomenology, “a method designed to understand people’s lived experiences and how they make sense of it in the context of their personal and social worlds”.^9^ (p. 3) This study was approved by the Wilfrid Laurier University research ethics board (REB#8744).

### Participant recruitment

Participant inclusion criteria are listed in Table 1. Participants were recruited between January and March 2024 from Southern Ontario using purposeful sampling, namely criterion and snowball sampling.^10^ When possible, participants were recruited as dyads (i.e., person with lupus and primary support person). However, one case was recruited as a triad, as the individual with lupus had two primary support persons; and one person with lupus did not have a primary support person who wanted to participate and thus was recruited individually.

**Table 1.** Inclusion criteria.

Inclusion criteria for individuals with lupus
- Clinical diagnosis of lupus
- Be a woman in university (e.g., currently completing an undergraduate degree, master’s degree, Ph.D., or other post-graduate university degree such as physiotherapy, occupational therapy) or graduated within the past 3 years
Inclusion criteria for support persons
- Perceived as a primary support person for the individual living with lupus
- Must provide or provided support to the individual with lupus during the university period

### Data collection & analysis

Following informed consent, each participant was emailed a background questionnaire to assess eligibility and to provide context prior to the one-on-one semi-structured interviews. Interviews were conducted via Zoom (*n* = 9) or over-the-phone (*n* = 1). The interview lengths were dependent on the depth of participants’ responses (range = 35 to 102 min). All participants were interviewed individually. The interview guide for the participants with lupus included questions concerning sociodemographic information, diagnosis, symptoms and treatments, and experiences navigating university and support services. Primary support persons’ interview guide included sociodemographic information, information about the participant with lupus, and their supportive role. All interviews were audio recorded and then transcribed verbatim using Microsoft Word software. Pseudonyms were utilized to protect the anonymity of each participant, while other identifying information was removed from transcripts. Braun and Clarke’s,^11^ six steps to reflexive thematic analysis were used to identify, analyze, and report themes and sub-themes within the data collected (see, Braun & Clarke, 2019^11^ for more detailed information on this analysis). Aligning with the theoretical orientation of interpretive phenomenology, analysis began with an idiographic focus, whereby each author read and re-read each transcript to gain an in-depth understanding of each participants lived experience, prior to identifying patterns across cases. Primarily inductive coding techniques were implemented, and themes were generated interpretively. All participants lived experiences, and the reflexive role of each author was considered throughout. This process contributed to the credibility of the results. Multiple modes of data collection (field notes, member checks, triangulation, reflexive journaling) were also utilized to promote credibility and confirmability.^12^

## Results

We interviewed five women with lupus and five primary support persons (*n =* 10). Three of the women with lupus were current university students (e.g., undergraduate, masters, post-graduate degree) and two were recent graduates (i.e., within 3 years). Four primary support persons were women, and one was a man; relationships to the individual with lupus varied (See Table 2).

**Table 2.** Participant demographic information.

Demographic information for individuals with lupus						
Participant	Age	Age at diagnosis	Type of lupus	Education status	Other health complications	Primary support person(s)
“Mia”	21	19	Probable lupus nephritis	Current 4th year undergraduate	Kidney disease	“Jane”
“Tessa”	25	18	Drug-induced	Recently graduated within 3 years from doctorate program	N/A	“Melissa” and “Thomas”
“Sarah”	24	14	SLE	Current post-grad college degree	N/A	“Leah”
“Victoria”	23	7	SLE	Current 3rd year undergraduate	Antiphospholipid anti-body syndrome, hypertension, Raynaud’s syndrome, rheumatoid arthritis, heart failure, CKD, kidney failure, and chronic skin rash	“Evelyn”
“Lauren”	26	10	SLE	Recently graduated within 3 years from Master’s program	N/A	Did not interview

Demographic information for support persons						
Participant	Age	# of children	Employment status	Marital status	Perceived health	Relationship to individual with lupus
“Jane”	46	1	Full-time	Single	Good	Mother of “Mia”
“Melissa”	57	4	Retired	Married	Good	Mother of “Tessa”
“Thomas”	26	0	Full-time	Engaged	Excellent	Fiancé of “Tessa”
“Leah”	56	2	Stay-at-home	Married	Good	Mother of “sarah”
“Evelyn”	54	2	Part-time	Married	Good	Mother of “Victoria”

Two salient themes were identified from data analysis: (1) Lupus is a part of me and (2) Lupus does not exist in a bubble. Themes and their respective sub-themes are outlined in Table 3 and discussed below.

**Table 3.** Themes and sub-themes.

Theme	Sub-theme
1. Lupus is a part of me	- The unpredictability of living with lupus
	- Ebbs and flows
	- All about perspective
2. Lupus does not exist in a bubble	- Lack of knowledge and awareness
	- “Advocacy. A lot of advocacy”

### Theme 1: Lupus is a part of me

This theme discussed how lupus was part of participants everyday lives and how it became their new normal following the onset of symptoms. From this theme, three sub-themes were evident. The first sub-theme, “The unpredictability of living with an individualized illness” discussed the differences among participants shared experiences either living with lupus or as primary support persons. The second sub-theme, “Ebbs and flows”, addressed how participants’ lupus fluctuated at different points in time and how it affected their lives. The last sub-theme, “All about perspective”, focused on participants’ initial reactions to the diagnosis, shifts in attitude, and lessons learned from living with lupus or providing support. Each sub-theme is described below, and illustrative quotes for each sub-theme are listed in Table 4.

**Table 4.** Themes, sub-themes, and illustrative quotes.

Themes	Sub-themes	Illustrative quotes
Lupus is a part of me	The unpredictability of living with lupus	“The prednisone. That’s the one that made me gain 20 pounds of water weight …. that disabled me physically …. walking up the stairs required support from my mom as it felt like I was lugging sandbags on my feet, and I often felt out of breath at the top of the stairs. I could dress myself, eat, walk, sit, and stand, but everything I did felt more difficult and like I was moving very slow. In the hospital specifically, I recall my mom helping me bathe since I had some joint-pain, and she was probably worried I would slip in the shower.” - Sarah PwL
		“Once I started taking prednisone, and I started seeing myself blow up into that big puffy balloon …. I kind of lost a sense of identity, because I started going back to school at [university A], and my friends weren’t even recognizing me. And I was like, this really sucks [emphasis] …. it was sad.” – Tessa PwL
		“Socially it has affected me because I created a lot of friendships going into university, I met a lot of people, but I would drop off for weeks at a time because of how I was feeling. [People] are going through their own stuff. They’re in the middle of studying; they’re in the middle of going to parties …. so you kind of get pushed to the wayside a little bit and you don’t get to enjoy those friendships as much as you could if you were able to just go out all the time, or study together all the time, or just be present in class for them to sit next to you and talk to you.” – Victoria PwL
		“I had to put dance on hold because I wasn’t physically well. So that was a challenge because I missed my dance friends. I missed being able to go to dance every day. I didn’t get the chance to have a normal first year of high school. So, I feel socially, the diagnosis really affected me because my friends made new friends, … I definitely struggled emotionally with that – Sarah PwL
		“I’ll always call my mom … just talking to her about her day, that just kind of distracts me from what’s going on with me. So that makes me feel better” – Mia, PwL
		“…. I try my best to keep upbeat about things, to keep positive and just say, ok, if this didn’t work out, I’ll do something else. I also try to find joy in my hobbies, the things that I like to do at home by myself, so that I don’t feel like I’m missing out on life itself. I just may be missing out on something that’s going on outside.” – Victoria PwL
	Ebbs and flows	“[a good day] would be nothing at all …. no inflammation, no change in vision, no brain fog, and full energy” – Tessa PwL
		“It has shifted … At the beginning of my diagnosis, the first 3 years, she [my mother/support person] was definitely more there for me health wise, making sure I [was] attending all my appointments …. also just making sure I was wearing sunscreen or…. eating healthy …. But now that I’m older, I’ve lived by myself, I’m more aware of my symptoms, what to look out for in case I get a flare up …. I’m also responsible for taking myself to my appointments now …. She’s still there emotionally, medically, but not as much, which I think is good because it taught me to take care of myself.” – Sarah PwL
		“At school it’s honestly a little bit more difficult because she’s [my mother/support person] not physically here …. at school, it’s just daily FaceTime calls just to make sure that I’m ok and distract me a little bit …. when I’m at home, she definitely has a more hands on role. She’ll make sure that I’m not exasperating myself in any type of way, she wants me to just chill when I’m at home because she knows when I’m at school, I’m doing everything myself” – Mia PwL
		“…. But I think stress is a huge factor [effecting my lupus]. You know, like school demands, more assignments, wondering if I am going to pass or not” – Sarah PwL
		“I had to drive to campus every single day. So there were days where either it was too snowy and I was like, I don’t trust my eyes [because they got blurry during a flare], so I wouldn’t go” – Tessa PwL
	All about perspective	“Honestly, I was really sad. I think I went through a little phase, I wouldn’t say it was depression, but I went through a phase of the unknown, because there’s not much research out there. There isn’t. And that was the scary part, when you’re looking things up when you’re first getting diagnosed, it was like, kidney failure, liver failure, your organs will attack itself … I was just healthy not even 2 weeks ago, working out at the gym and being a good student and hanging out with friends, and now here I am in a completely different stage of life. So, I think it was just a huge shock.” – Tessa PwL
		“I was relieved a little bit, to be able to be diagnosed with something, or at least have more continuity of care …. I was just excited to no longer have to go to a bunch of doctor’s appointments, it [went] from all these different dermatologists, to now just once a month down at [hospital E], which felt much more manageable” – Lauren PwL
		“Scared. Scared was the biggest thing …. because you can’t change it, right? You can’t change it. All you can do is try to work around the symptoms and try to keep her as healthy as you can. You can’t take it away from her” – Melissa SP
		“I told [my daughter] that it was a lifestyle change …. and that [she is] not on [her] deathbed. And I think to come at it from that perspective, was better than the alternative” – Jane SP
		“Believe in yourself, because there is always, always a light at the end of the tunnel. And whatever you do, try not to label yourself as lupus. Because that’s one thing I started to do I was like, ohh I have lupus and it would become my identity … be yourself regardless, but always listen to your body, your body knows best.” – Tessa PwL
		“I think with a chronic illness in university …. figure out what matters most to you. Is it academics? Is it social? Is it perspective, job planning, or your future, and prioritize accordingly. And there is absolutely nothing wrong with whatever you choose to prioritize. So, if that’s going to [club] on a Sunday …. if that means you have to sleep in and miss class, I don’t think that there’s anything wrong with that, as long as you’re ok with that decision.” – Lauren PwL
Lupus does not exist in a bubble	Lack of knowledge and awareness	“She was constantly seeing a different doctor every day and would have to repeat the story …. They were always misinformed, and I was always having to explain to them what was happening. And the nursing staff that were on the unit were very junior and weren’t taking things seriously. So, when [Tessa] was having an episode where she could barely walk, she couldn’t even stand on her feet, I had to carry her to the bathroom, I called the nurse and I said she was having problems walking, and they just said, well, she’s just got to push herself.” – Melissa SP
		“School support was probably the worst …. [Department Chair] wanted to actually kick me out of the program before I was even back from the hospital. He [said] ‘You need to drop out. You’re missing too much school’. [He] could not even understand where I was coming from …. I think the school support resources, as much as they want to help, they just absolutely have no clue. [They] literally have no clue …. that was one of the hardest things to deal with …. don’t tell me what to do when you have no clue. You don’t even know what lupus is.” – Tessa PwL
		“I actually haven’t found any resources at all at [university X] that have anything to do with lupus or kidney disease, or anything like that, which is kind of frustrating because I know it’s not a very common thing, but there’s just no support available for that specifically. And with the [university X accessible learning services], the process just seems so long that I never ended up doing it.” – Mia PwL
		“I was like, I don’t know why you guys are asking for more documentation every single year, when lupus is a systemic disease. This is ongoing [emphasis]. If you have a positive ANA, you have all these things, you have lupus [emphasis]. This is a lifetime diagnosis, stop asking me for stuff every single year. That was just frustrating because that’d also put on a financial burden, because then you were also asking your doctor to write these letters that were $50 each time” – Tessa PwL
		“It was frustrating …. it’d be different if she was just coming and saying, I need this accommodation …. she had heart surgeons [and nephrologists] that told her that she needed that accommodation. These are people who know what they’re talking about, right? They’re not making this up because she wants to finish a paper …. It was just so many hoops. Who’s got the energy to do that when you’re sick? …. It’s like you’re punishing the person who’s ill for being ill …. There’s no way that you should need to, in addition to learning, have to navigate this complicated process of having people try to believe that you have what you have.” – Evelyn SP
		“I didn’t lose friends from it [lupus], but I became less close with friends because of the diagnosis, mainly because people just didn’t understand. They were like, she’s so flaky, and at that point I was just like, ok well I don’t want to hang out with you then if you don’t understand” – Tessa PwL
		“I think stigma wise they [my friends] have been more ignorant” – Victoria PwL
	“Advocacy. A lot of advocacy”	“As a mother, you have to follow your instinct. I knew that there was something wrong and I brought her to the hospital. I think just making sure that if you’re not getting the right answers, that you need to seek them out, which is what we did. But some people don’t have anybody to advocate for them. They would have just went home and, you know, just got sicker and sicker, rather than figuring out what was wrong” – Melissa SP
		“Number one, is advocate for yourself. I know that as a young child, I didn’t have to, but I had a really strong mother who, to this day, continues to advocate for my health and well-being. And my original perspective was that, oh, the doctors know best, the doctors are always right, and you should always trust the doctors, and that’s not true. I’ve learned that [emphasis] many times over that it's not true. Doctors make mistakes too, because they’re human. And if you notice something’s wrong, or if you feel that you’re not being cared for correctly, speak up, because that could be the difference between you having worse symptoms, you having to do a whole procedure. And it’s just way better to stand up for yourself in that initial moment.”– Victoria PwL
		“I think just being more vocal about the diagnosis, because not a lot is known, and because, again, everyone experiences lupus so differently. And so, just because someone knows someone who has it, it doesn’t mean the next person they meet will fit in that same box. So, I definitely say to advocate for myself more and … be more vocal about how people can support me … looking back, it would have avoided a lot of awkwardness and would have made a huge impact on me emotionally and psychosocially, if I just would have taken that initiative to advocate for myself”” – Lauren PwL
		“What qualifies you to be able to get accessibility learning. I just never knew that I could be qualified to get those types of accommodations. So, I think also maybe offering support groups too would be really nice, because I haven’t met anyone else that has lupus that is my age – Mia PwL
		“I think one major thing is advertising more that there are different types of accessibility services. Because honestly, beyond note takers and I guess the accommodation residence stuff which I applied for … I just feel like it’s poorly advertised on the websites and also on campus. So, I think they could really improve on that … I feel like depending on some profs, some are more accommodating than others, and maybe others they don’t understand the extent to why you need that time off or something. So, I think there also needs to be education on professor sides, and understanding, you know, why does the student need this type of service.” – Sarah PwL
		“The main thing is to make sure that students who do have chronic illness have access to these supports … I do worry that people who have chronic illness aren’t accessing these, and I can’t imagine having to go through university without that. Um, I don’t know what that looks like though. I think it’s being a little bit more creative with the documentation that’s required to be able to access those services. And then the promotion of it” – Lauren PwL
		“Definitely having more educated people in the [university support service]. The learning centre definitely needs to have people … systemic illness is completely different [emphasis] than other illnesses. Not to say those aren’t systemic either, like anxiety, depression, PTSD, things like that. But I think that there’s fine lines between all these diagnoses, and someone with say, even just diabetes versus SLE, they’re so different [emphasis]. And I think that there’s so many different people, and everyone has a different story, everyone has different symptoms, and I think schools need to have people who have more education on things like that” – Tessa PwL

#### The unpredictability of living with lupus

Participants described lupus as an inherently individualized condition, whereby no two experiences with the disease are the same. While there were some shared symptoms (e.g., fatigue), differences in diagnosis journeys, symptom presentation, and disease progression contributed to an overall agreement that lupus is unpredictable.

For starters, the unpredictability associated with lupus was reflected in participants diagnosis journeys, which were varied considerably in both timing and process. For example, two participants were diagnosed with lupus early in childhood, one participant during adolescent years, and two during their undergraduate degrees. However, regardless of timing, most participants (*n =* 4) described the diagnostic process as challenging, as it involved being misdiagnosed or feeling dismissed by healthcare providers. The individual diagnostic pathways shaped how participants made sense of their illness, as those who were misdiagnosed or dismissed by healthcare professionals reflected on prolonged feelings of uncertainty and frustration which in turn affected other aspects of their lives (e.g., academics, social life, etc.).

Variability was also evident in symptom presentation and severity across participants. Although all participants with lupus experienced fatigue (*n =* 5), other symptoms (e.g., hair loss, swelling, neurological deficits), the severity of symptoms, and the development of additional health complications (e.g., high blood pressure, Raynaud’s kidney issues, heart issues) varied across participants. Participants described how this variability extended beyond physical symptoms and influenced their mental health, social lives, ability to participate in sports and physical activity, relationships, and overall university experiences. As such, due to the unpredictable and ever-changing nature of their lupus, participants expressed an overarching lack of control over their bodies and daily functioning.

The individualized nature of lupus was further reflected when discussing treatment and general effects of the disease. For example, Sarah and Lauren discussed the effects of prednisone and hydroxychloroquine and described how treatment related side effects impacted their independence and ability to complete daily activities. Likewise, Tessa and Victoria highlighted how their comorbid health complications restricted their freedom, such as their ability to be outside, travel, and consume what they wanted. These experiences highlighted how lupus manifested in distinct ways, reinforcing the sense that each participant was navigating a unique illness trajectory.

Lastly, due to the varying effects experienced by all participants with lupus, participants implemented varying coping strategies to manage their health and monitor symptoms. Among the most common were diet (*n =* 3) and exercise (*n =* 5). Other strategies included buying hair extensions to hide the hair loss from treatment (*n =* 1), social support from friends, family or therapists (*n =* 2), avoidance (*n* = 1), and positive thinking/self-talk (*n* = 1). The diversity of these strategies reflected participants’ ongoing efforts to adapt to an illness that was unpredictable, requiring continual adjustment to their changing physical and emotional needs.

#### “Ebbs and flows”

“Ebbs and flows” or the continuous change occurring was consistent among participants’ lupus experiences. For example, participants described intermittent symptoms throughout their disease course when differentiating between “good days” and “bad days”. On good days, most participants described having relatively no symptoms and “feeling normal” (Mia). In contrast, participants described bad days as feeling extremely fatigued (*n =* 5), unable to get out of bed (*n =* 5) and having difficulty completing tasks (*n =* 5). Victoria summarized the stark difference between the two as follows: “You can catch me on a good day, or a really bad day, and you’ll see two completely different people”. Participants with lupus also described exacerbating factors (e.g., stress, weather) which often contributed to severe flare-ups. These flare-ups affected participants with lupus ability to attend classes as participants described the lack of energy to drive or walk to campus (*n =* 4) or an inability to focus due to pain (*n =* 3). During the interviews, participants mentioned they were either experiencing periodic mild symptoms or were in complete remission, further highlighting the ebbs and flows of lupus.

Ebbs and flows of support from primary support persons was also evident during active and inactive periods. For example, Sarah talked about the difference in support she received from her mother during her diagnosis, in comparison to when she had been living with lupus for a longer period of time. Two other participants (Lauren & Mia) shared the lessened support from their mothers while at university compared to at home.

In sum, the ebbs and flows of the participants’ lupus experiences as evidenced by their fluctuating symptoms and the varying levels of support received made daily living and disease management intermittently unpredictable and difficult to navigate.

#### “All about perspective”

This sub-theme discussed participants with lupus and their primary support persons shift in perspective following the diagnosis. Specifically, all participants shared their shift to more positive outlooks when referring to their diagnosis, proposed advice for others in similar situations, and overall reflections on their lupus experiences. When participants with lupus were asked about their initial reactions to their diagnosis, responses varied. Some participants with lupus described feeling shocked (*n* = 3) and heartbroken (*n* = 2), whereas others were naive to the severity of the disease (*n* = 2) or relieved to finally have a diagnosis (*n =* 1). Reactions of primary support persons also varied. Some primary support persons described feeling scared (*n* = 3) or frightened due to a lack of understanding of what the future would like for the person with lupus (*n* = 2). However, after processing the diagnosis, all participants expressed a shift to more positive outlooks. For example, Jane who was originally fearful for her daughter’s future, later stated lupus should just be viewed as a lifestyle change. Other participants felt similarly after being educated on what having lupus really meant.

Due to the shift in mindset, participants were able to identify other positives that emerged because of lupus. For example, participants with lupus described living healthier lives (*n =* 3) and being more in tune with their bodies (*n* = 1). Likewise, participants described ways lupus contributed to their personal growth, such as knowing when to ask for help (*n =* 5) and enhanced self-confidence (*n =* 1), knowledge (*n =* 5), and independence (*n =* 1). Primary support persons were also able to identify positives from lupus. One primary support person indicated she was more appreciative when spending quality time with her daughter, while others shared feeling grateful for their own health and the importance of living a healthy lifestyle.

By reflecting on their experiences with lupus, participants were able to share advice for others currently seeking a diagnosis or navigating their own journeys with lupus. Most participants recommended taking it day by day (*n* = 3), being kind to oneself (*n* = 2), and surrounding oneself with supportive people (*n =* 4). One participant with lupus (Lauren) specifically tailored her advice to university students and emphasized the importance of prioritizing time and energy. Primary support persons also shared advice for others, and all emphasized the importance of leaning on others and asking for help when needed (*n* = 5).

### Theme 2: Lupus does not exist in a bubble

This theme discussed the external factors that impacted the experiences of the participants with lupus as well as the influence lupus had on others. From this theme, two sub-themes developed. The first sub-theme, “Lack of knowledge and awareness”, discussed the challenges individuals with lupus and support persons experienced within healthcare, education and society. The second sub-theme “Advocacy. A lot of advocacy” provided advice for others living and caring for lupus and the importance of advocating within healthcare and education. Like theme 1, each sub-theme is described below, and illustrative quotes are listed in Table 4.

#### Lack of knowledge and awareness

Regardless of geographical location, participants with lupus and support persons experienced a lack of knowledge and awareness when interacting with healthcare professionals, educational staff and friends, negatively influencing their experiences with lupus. Three participants with lupus (Mia, Victora & Sarah) described having their symptoms and concerns dismissed by healthcare professionals. For one participant (Victoria) this led to emergency heart valve replacement surgery as the doctors dismissed the severity of her flu-like symptoms. One primary support person (Melissa) described her frustration due to the lack of knowledge her daughter’s nurses possessed when she was admitted to the hospital during a flare. Similar experiences were described by other support persons (*n* = 3).

Participants with lupus and support persons expressed noteworthy challenges within university due to professors and support staff not recognizing the complexities of lupus. For one participant (Tessa) the chair of the department wanted to kick her out of her program for missing too much school despite being in the hospital. This lack of regard from university staff was experienced by other participants as well (*n* = 3). To help manage their lupus while at university, some participants searched for support groups to join but were unsuccessful (*n =* 2). In terms of academic accommodations, some participants (*n =* 2) were unaware they qualified due to the language used by learning support centres. Other participants expressed the burdensome process associated with seeking accommodations deterring them from getting the support they needed (*n =* 2). Participants with lupus and support persons discussed how the invisible nature of lupus elevated their challenges when seeking support. Two participants (Victoria & Tessa) were fortunate to attend universities with exceptional accommodation services and described how beneficial this was for them. Specifically, Tessa discussed the stark difference between her experience in her undergraduate degree compared to graduate school, as she went from being disregarded with no accommodations to being fully accepted and supported.

Participants also discussed challenges due to the lack of knowledge friends and family members had regarding the needs of someone living with lupus. All participants with lupus described challenges during university as they could not socialize (e.g., go out for dinner, go to a party etc.) with their friends due the limitations (e.g., dietary restrictions, fatigue) they experienced from lupus. Participants with lupus also experienced challenges with family members as they would continuously forget about their dietary restrictions (*n* = 1) or not understand why they were tired. This caused participants with lupus to feel isolated and alone.

#### “Advocacy. A lot of advocacy”

Due to the lack of knowledge and awareness surrounding lupus, participants with lupus and primary support persons emphasized the importance of advocacy and fighting for their needs within the healthcare and education system. Specifically, support persons (Melissa & Evelyn) stressed the importance of advocating for their daughters at medical appointments and advised healthcare professionals to trust the parents because they know their children best. For participants with lupus (*n* = 2) they reminisced about their early days of diagnosis and wished they advocated harder for themselves and thus advised others living with lupus to not be afraid to question their doctors.

Outside of a healthcare setting, participants also emphasized the importance of advocacy within university/college. Within the interviews participants with lupus and support persons were asked to provide advice for others living or caring for lupus or advice for the education system regarding support for individuals with lupus. Consistent with the advice for others within healthcare, all participants (*n =* 10) advised people with lupus to be vocal and advocate for their needs when interacting with professors and other education staff, as this is something they had not always done for themselves. Specifically, support persons (*n* = 3) stressed how they wished they advocated harder for the individuals living with lupus so that they would have received more support by the university/college. In terms of the advice for universities themselves, all participants indicated the need for increased awareness and better advertising strategies. Two participants (Mia & Victoria) advised universities to form support groups for students living with chronic illness to help connect students and make them feel less isolated. Other suggestions included simplifying the application process for accessible learning services (*n* = 3) and educating professors and accessibility staff on the needs of students with chronic illnesses, such as lupus (*n* = 2).

## Discussion

This study explored the lived experiences of young adult women navigating university/college and their primary support persons to better understand the effects of navigating through this transitional period. Results highlighted although all participants’ journeys with lupus were unique, they experienced similar challenges such as extreme fatigue and pain interfering with their ability to attend classes and socialize with others while at university. Likewise, several participants reported inadequate support while at university due to professors and other educational staff not recognizing the needs of individuals living with lupus. The lack of knowledge about lupus was also evident within healthcare further exacerbating the effects experienced by all participants. To help mitigate the effects experienced by other university students with chronic illnesses, participants recommended universities simplify the process associated with applying for academic accommodations and offer support groups to help connect those with similar experiences.

To our knowledge, this is one of the first studies to explore the experiences of women navigating university/college and their primary support persons. Several other studies have explored the experiences of younger individuals with lupus within elementary school,^2,4^ or high school^2,4^; however, limited studies have focused on those within university/college. Agarwal and Kumar,^6^ is one of the only other studies to focus on this population. The results of our study were consistent with Agarwal and Kumur emphasizing the varying needs of persons living with lupus despite having the same diagnosis. Agarwal and Kumur also provided implications for universities which consisted of offering support groups for students living with lupus and increasing awareness and understanding of lupus for professors, students, and other educational staff. However, it is important to recognize Agarwal and Kumur conducted a singular case study and thus only considered the experiences of one student living with lupus. As such, our study adds to the current knowledge base regarding the experiences of those living with lupus within university/college and may be used to (1) help educate professors, students, and other staff on the needs of students living with lupus and (2) how to adjust accommodation services to better meet the needs of students living with invisible chronic illnesses. Although our study builds on the results from Agarwal and Kumur,^6^ future research should continue to explore university aged students with lupus to aid in service development and enhancement for this population.

In addition to interviewing university/college aged women with lupus we also interviewed their primary support persons. From our understanding this is one of the first studies to consider the role of primary support persons in the context of university/college aged students with lupus; however, primary support persons have been explored within the context of other chronic illnesses.^13^ Primary support persons or caregivers serve as advocates and provide physical, emotional and financial support and are deemed essential for anyone living with a chronic illness.^13^ Within our study the role of primary support persons varied between participants; however, like the results of previous studies, they all served as a critical source of support for participants with lupus. Primary support persons emphasized being an advocate for their care recipients with lupus and wished healthcare professionals would trust their knowledge as they know their care recipients best particularly during diagnosis and early stages of disease navigation. The notion of healthcare professionals trusting or honouring the knowledge of primary support persons is seemingly novel within the context of individuals with lupus; however, has been discussed among other populations.^14^ Rao and Raj,^15^ explored this concept qualitatively from the perspective of twenty physicians. Results indicated establishing trust between caregivers and physicians is complex; however, physicians are more apt to trust caregivers when they demonstrate reliability, competence and fidelity.^16–18^ Nonetheless, the process of determining if a caregiving possessed these qualities was ambiguous and varied from physician to physician.^15^ Within our study, participants with lupus and primary support persons advised others to advocate for their needs and ask questions to ensure their concerns were not being dismissed as many participants did not believe their physicians valued or trusted their opinions. Future research should continue to explore the relationship between primary support persons (caregivers) and physicians to help ensure the voices of primary support persons are being heard.

Despite noteworthy contributions to the literature, this study is not without limitations. Although participants provided detailed accounts of their experiences with lupus the results of our study are not intended to be representative of all university/college aged students living with lupus or primary support persons. For example, our study was restricted to women and their primary support persons and thus the results may not be transferable to men living with lupus within university/college or primary support persons of men with lupus. It would be worthwhile for future research to explore the experiences of university/college aged men with lupus and their primary support persons to understand their experiences during this transitional period. Nonetheless, our study has several practical implications. Due to the paucity of information on university/college aged students with lupus the results of our study could be used to help educate and inform university/college staff and students on the effects experienced by students living with lupus. Further, the results of our study may help accessible learning centres within university/college refine their services to better meet the needs of students living with invisible chronic illnesses, such as lupus. Lastly, due to the challenges associated with navigating university life expressed by all participants it may be worthwhile for universities/colleges to have a primary point of contact for students with chronic illnesses to help mitigate some of the challenges associated with navigating university.

## Conclusion

This was the first study to consider the role of primary support persons in the context of university/college aged students with lupus. Our findings suggest women in university/college with lupus experience a variety of effects (e.g., physical, psychological, social) which are exacerbated due to the lack of knowledge professors, healthcare professionals, students, and other education staff possess regarding the needs of individuals with lupus. All primary support persons played an integral role due to the lack of external support provided within most university/colleges. Participants advised universities’/colleges’ to enhance advertisements/education and simplify the application process for accessible learning services and offer support groups for students living with chronic illnesses to more effectively navigate post-secondary education.

## Data Availability

The qualitative data supporting the findings of this study are not publicly available due to privacy restrictions.*
